# Medical Radiation Exposure Reduction in PET via Super-Resolution Deep Learning Model

**DOI:** 10.3390/diagnostics12040872

**Published:** 2022-03-31

**Authors:** Takaaki Yoshimura, Atsushi Hasegawa, Shoki Kogame, Keiichi Magota, Rina Kimura, Shiro Watanabe, Kenji Hirata, Hiroyuki Sugimori

**Affiliations:** 1 Department of Health Sciences and Technology, Faculty of Health Sciences, Hokkaido University, Sapporo 060-0812, Japan; takaaki.ysm@med.hokudai.ac.jp; 2 Department of Medical Physics, Hokkaido University Hospital, Sapporo 060-8648, Japan; 3 Department of Health Sciences, School of Medicine, Hokkaido University, Sapporo 060-0812, Japan; ha829handball3104@eis.hokudai.ac.jp; 4 Graduate School of Biological Science and Engineering, Hokkaido University, Sapporo 060-8638, Japan; shoki.kgm@frontier.hokudai.ac.jp; 5 Division of Medical Imaging and Technology, Hokkaido University Hospital, Sapporo 060-8648, Japan; magota@huhp.hokudai.ac.jp; 6 Department of Diagnostic and Interventional Radiology, Hokkaido University Hospital, Sapporo 060-8648, Japan; rinakimura343@gmail.com; 7 Department of Diagnostic Imaging, Graduate School of Medicine, Hokkaido University, Sapporo 060-8638, Japan; shirow@med.hokudai.ac.jp (S.W.); khirata@med.hokudai.ac.jp (K.H.); 8 Department of Nuclear Medicine, Hokkaido University Hospital, Sapporo 060-8648, Japan; 9 Department of Biomedical Science and Engineering, Faculty of Health Sciences, Hokkaido University, Sapporo 060-0812, Japan; 10 Clinical AI Human Resources Development Program, Faculty of Medicine, Hokkaido University, Sapporo 060-8648, Japan

**Keywords:** deep learning, PET, radiation exposure, super-resolution

## Abstract

In positron emission tomography (PET) imaging, image quality correlates with the injected [18F]-fluorodeoxyglucose (FDG) dose and acquisition time. If image quality improves from short-acquisition PET images via the super-resolution (SR) deep learning technique, it is possible to reduce the injected FDG dose. Therefore, the aim of this study was to clarify whether the SR deep learning technique could improve the image quality of the 50%-acquisition-time image to the level of that of the 100%-acquisition-time image. One-hundred-and-eight adult patients were enrolled in this retrospective observational study. The supervised data were divided into nine subsets for nested cross-validation. The mean peak signal-to-noise ratio and structural similarity in the SR-PET image were 31.3 dB and 0.931, respectively. The mean opinion scores of the 50% PET image, SR-PET image, and 100% PET image were 3.41, 3.96, and 4.23 for the lung level, 3.31, 3.80, and 4.27 for the liver level, and 3.08, 3.67, and 3.94 for the bowel level, respectively. Thus, the SR-PET image was more similar to the 100% PET image and subjectively improved the image quality, as compared to the 50% PET image. The use of the SR deep-learning technique can reduce the injected FDG dose and thus lower radiation exposure.

## 1. Introduction

Deep neural networks have been applied in computer vision tasks, such as segmentation, image classification, denoising, image generation, image synthesis, and super-resolution (SR). Among them, SR is one of the most popular approaches used for increasing the resolution of degraded low-resolution (LR) images. Many SR models have been published since the SR convolutional neural network (SRCNN) was first reported by Dong et al. in the European Conference on Computer Vision 2014 [1]. A summary of the SR challenge in the New Trends in Image Restoration and Enhancement workshop is reported annually [2,3,4,5,6]. Moreover, many open-access state-of-the-art (SOTA) models have been published on websites [7]. These technologies are attracting attention not only in natural image processing but also in medical image processing, and deep neural networks have been applied to nuclear medicine [8].

Positron-emission tomography (PET) is a functional imaging modality that uses radiotracers, such as [18F]-fluorodeoxyglucose (FDG), and has been used for the diagnosis of cancer and assessment of the extent of disease in oncology, combined with anatomical data from computed tomography (CT) or magnetic resonance imaging (MRI) [9,10,11]. However, one of the limitations of PET imaging is its relatively poor spatial resolution, as compared with CT or MRI, because of physical parameters, such as scatter, counting statistics, position range, and patient motion. Currently, a small number of clinical PET systems using silicon photomultipliers (SiPM) are commercially available, such as the Signa PET/MRI system (GE Healthcare, Waukesha, WI) [12], Discovery MI PET/CT system (GE Healthcare) [13], Vision PET/CT system (Siemens, Munich, Germany) [14], and Vereos PET/CT system (Philips, Amsterdam, Netherlands) [15]. These clinical PET systems achieve high-energy resolution (<10% in full-width at half-maximum (FWHM) and precise time-of-flight (TOF) measurements (<400 ps FWHM in coincidence time resolutions). Although the PET systems equipped with SiPM are expensive, the use of these systems is expected to become widespread in the future because they can improve the image resolution as compared with the conventional PET system without increasing radiation exposure for the patients [12,13,14,15,16,17].

In Japan, radiation exposure management has been obligatory since April 2020, due to the enforcement of the partial revision of the Enforcement Regulations of the Medical Care Law, which includes the safety management of radiation for medical use based on the established Japanese diagnostic reference levels (DRLs) 2020 [18]. Abe et al. reported the details of Japan’s DRLs 2020 for nuclear medicine [19]. Depending on the examination protocol, the radiation exposure in FDG-PET/CT imaging is higher than that in contrast-enhanced CT imaging [20,21]. Thus, the absorbed radiation dose per examination should be as low as possible, particularly for young patients who are sensitive to radiation and potentially require repeated follow-up studies. Previously, Queiroz et al. reported that the quality of PET images with half the injected FDG dose was clinically acceptable [22]. In addition, Sekine et al. demonstrated that the PET image quality in a TOF PET/MR system was clinically adequate with 60% of the usually injected FDG dose in patients with a body mass index (BMI) > 25 kg/m^2^ and 50% of the injected FDG dose in patients with a BMI < 25 kg/m^2^ [23].

The noise-equivalent count correlates with the acquisition time and injected dose in FDG-PET. At the point of radiation exposure, a reduced dosage of injected FDG is desirable for patients. However, PET images with a decreased signal-to-noise ratio (SNR) and structural similarity (SSIM) would affect disease diagnosis. In clinical practice, diagnostic examinations are performed based on defined procedure guidelines for tumor imaging [20,24], so it is not easy to obtain images with low-dose FDG injections for ethical reasons. Thus, it is important to simulate low-dose data from the under-sampled normally injected FDG dose, for further injected FDG dose reductions.

We hypothesized that it would be possible to reduce the radiation exposure in PET examinations by improving the image quality via SR deep learning techniques from low-quality PET images obtained with short acquisition times. Thus, the aim of this study was to clarify whether, when using a short acquisition time, PET image quality could improve to a level similar to the conventional full-acquisition-time PET image by applying the SR deep learning model.

## 2. Related Works

SR techniques have been applied in PET imaging to solve the problem of blurry reconstructed PET images due to noise and artifacts. The traditional SR approach is based on interpolation such as the most commonly used bilinear or bicubic methods. These interpolation methods increase the number of pixels and improve the image resolution with the obtained polynomial function constructed from all known points. Since the publication of SRCNN, research on SR using convolutional neural networks (CNNs) has dramatically advanced, and various deeper network architectures have been proposed [1,2,3,4,5,6,7]. These SR models succeeded in achieving SR with higher accuracy than previous interpolation methods. Recent SOTA methods with SR deep learning techniques have shown exceptional performance for natural images [2,3,4,5,6,7]. Following the Preferred Reporting Items for Systematic Reviews and Meta-analyses (PRISMA) standard, Ooi et al. systematically reviewed the SR deep learning algorithms [25]. Although unsupervised learning models such as the generative adversarial network (GAN) have also been proposed [26], most of these methods are based on the framework of supervised learning. These supervised learning SR frameworks artificially create the under-sampled low-resolution images from the given ground-truth high-resolution images and are trained to recover the original ground-truth images from the low-resolution images. Previously, image-generation techniques using GAN, such as PET images at normal doses being generated from reduced doses, have been considered to reduce radiation exposure in nuclear medicine [27,28]. On the other hand, the use of SR deep learning was limited for the purpose of radiation exposure reduction in PET imaging.

## 3. Materials and Methods

### 3.1. Patients and Image Acquisition

This retrospective observational study was conducted according to the guidelines of the Declaration of Helsinki and approved by the institutional review board (approval No. 020-0070). The need for written informed consent was waived due to the retrospective nature of the study. All images were acquired using the Vereos PET-CT system at our institution between April 2019 and May 2020. A total of 108 adult patients, 108 examinations, and 25,678 PET images were enrolled in this study (Table 1). The number of images varied from patient to patient (Table 1). No more than one whole-body scan was performed on each patient. All patients fasted for ≥6 h before FDG injection (ca. 4 MBq/kg), and emission scanning was initiated approximately 60-min post-injection. The effective dose was used to calculate the whole-body dose to compare the radiologic detriments from different radiation exposures. The effective dose from 18F-FDG PET scans was calculated as the product of injected 18F-FDG radioactivity and the dose coefficient weighting factor recommended in the International Commission on Radiological Protection publication 80 [11,29]. This weighting factor was set at 7.0 mSv/MBq for adults when 18F-FDG was administered to be 370 MBq. All images were reconstructed using an ordered-subset expectation maximization (OSEM) algorithm, time-of-flight algorithm, and point-spread function correction. The reconstructed images had a matrix size of 144 × 144 and voxel size of 4.0 × 4.0 × 4.0 mm. In this study, the “ground-truth” PET images in each patient were reconstructed at 90 s, which unified the collection time in all patients. To obtain PET images with simulated reduced injected FDG doses, three types of short-acquisition-time PET images (10%, 20%, and 50%) were reconstructed from identical PET emission data for each patient.

### 3.2. Super Resolution

**Figure 1:**
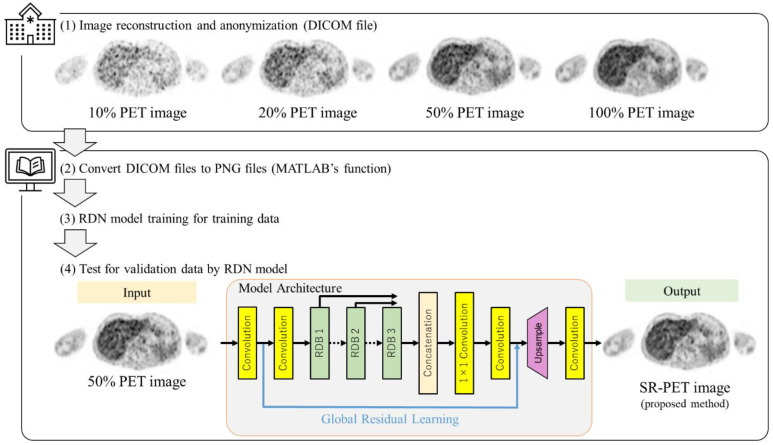
Flow chart of the proposed method and model architecture in this study. DICOM: Digital imaging and communications in medicine, PET: Positron emission tomography, SR-PET: Super-resolved PET, RDN: Residual dense network [30], RDB: Residual dense blocks.

We used the residual dense network (RDN) model for this study, which acquired SOTA at the time of research planning [30]. In short, this SR model fully exploited the hierarchical features from all convolutional layers by the residual dense block (RDB), which allowed direct connections from the state of the preceding RDB to all layers of the current RDB. Consequently, the RDN model achieved better/comparable performance against SOTA in experiments on benchmark datasets with different degradations [30]. This SR model was limited to importation in an 8-bit 3-channel RGB color image. Because the PET images represent one gray-scale channel, the training data in one channel were trained and concatenated with those in the other two channels to construct complete, color, high-resolution images. Thus, all reconstructed PET image data were anonymized and converted from 16-bit grayscale digital imaging and communications in medicine (DICOM) files to 8-bit three-channel grayscale portable network graphic (PNG) files using MATLAB’s (MATLAB2019b, The MathWorks, Natick, MA, USA) “mat2gray” function. We used a computer with two graphic processing units: NVIDIA GeForce GTX 1080 Ti 11GB (NVIDIA Corporation, Santa Clara, CA, USA). In the RDN model training, because the RDN model does not require low-resolution images, training data of the full-acquisition-time PET images (ground-truth) were used, and one-fourth were downsampled with the bilinear method. After the training, the predicted SR image was upsampled 4 times from the low-resolution input test images, which was not included in the training data. The training model hyperparameters were as follows: Maximum number of training epochs, 100; initial learning rate, 10^−5^; mini-batch size, 4. We divided the reconstructed PET images into 9 equal subsets according to the number of patients. Based on the subsets, we performed a 9-fold cross-validation procedure. We imported the half-acquisition-time PET image (50% PET image) as the LR image to the RDN and subsequently predicted the SR image. Overall, the output SR image had a four-fold upscaling resolution from these 50% PET images. Figure 1 shows the flow chart of the proposed method and the model architecture in this study.

### 3.3. Evaluation

We divided the supervised data into nine subsets for nested cross-validation [31]. Each subset was an independent combination of 96 patients used for training and 12 patients used for test images to prevent the overlap of patient images between the training and testing images within the subsets. To determine the effectiveness of SR for radiation exposure reduction in PET examinations, we performed objective and subjective evaluations. The objective evaluation is aimed at determining whether the SR model can be used to approximate the reference image better, and the subjective evaluation is aimed at determining whether the SR model can achieve an image quality appropriate for diagnosis.

For objective evaluation, 50% of the PET images were first downsampled using a bilinear model with a scaling factor of 4. Next, downsampled 50% PET images were upsampled by bilinear and RDN models with a scaling factor of four, to compare the output SR image and original “ground-truth” images in the same matrix size. The peak signal-to-noise ratio (PSNR) and structural similarity (SSIM) were calculated using MATLAB “PSNR” and “SSIM” functions [32,33]. These objective indices are well-known quality metrics for the comparison of two images. The PSNR is based on comparisons using explicit numerical criteria, using the mean squared error (MSE). However, SSIM is considered to be correlated with the quality perception of the human visual system, designed by modeling any image distortion as a combination of three factors (loss of correlation, luminance distortion, and contrast distortion). For a reference image x and test image y, the details of the PSNR and SSIM calculation equations are as follows:

(1) $$PSNR\left(x,\;y\right)=10\;log_{10}\frac{V_{p}{}^{2}}{MSE\left(x,\;y\right)}$$

(2) $$MSE\left(x,\;y\right)=\frac{1}{M\;N}\overset{M}{\underset{i=1}{\sum }}\overset{N}{\underset{j=1}{\sum }}{\left(x_{ij}-y_{ij}\right)}^{2}$$

(3) $$SSIM\left(x,\;y\right)=\frac{\left(2\mu_{x}\mu_{y}+C_{1}\right)\left(2\sigma_{xy}+C_{2}\right)}{\left(\mu_{x}^{2}+\mu_{y}^{2}+C_{1}\right)\left(\sigma_{x}^{2}+\sigma_{y}^{2}+C_{2}\right)}$$

(4) $$C_{1}={\left(0.01\;L\right)}^{2}$$

(5) $$C_{2}={\left(0.03\;L\right)}^{2}$$

where $V_{p}$ is the peak value of the signal, which was set to (2^8^ − 1 = 255) in this study; MSE(x, y) is the simplest and most widely used full-reference quality metric calculated by averaging the squared intensity differences of distorted and reference image pixels; $\mu_{x}$ and $\mu_{y}$ are the means of x and y; $\sigma_{xy}$ and $\sigma_{y}$ are the variances of x and y; $\sigma_{xy}$ is the covariance of x and y; and L is the dynamic range of the pixel values (255 for 8-bit grayscale images). The value of PSNR(x, y) approaches infinity as MSE(x, y) approaches zero, and a small value of PSNR(x, y) implies large numerical differences between x and y. The SSIM ranges from 0 to 1, where 1 denotes perfect similarity between two images. These measures were calculated for all images.

For the subjective evaluation, 50% PET images were upsampled using the RDN model. Bilinear interpolation was used to resample the other PET images to the same matrix size as the output SR-PET images. Three experienced board-certified nuclear medicine physicians (KH, 14 years; SW, 4 years; and RK, 1 year after board certification) visually evaluated all the images, independently, without access to the image label (i.e., which recovery method was used). We selected images for the subjective evaluation of the lung, liver, and bowel levels of each patient. All images were provided to the physicians in random order for image review. We performed a mean opinion score (MOS) test to quantify this ability [34]. Specifically, we asked these physicians to assign scores from 1 (poor image quality) to 5 (excellent image quality) to the original PET image, 10% PET image, 20% PET image, 50% PET image, and image super-resolved via RDN from the 50% PET image. Intraclass correlation coefficients (ICCs) were used to assess agreement between quantitative measurements in terms of consistency and conformity [35]. Based on ICC selection guidelines, we used the following forms using two-way mixed effects for model selection, consistency for definition selection, and single rater for type selection [36]:

(6) $$ICC\left(3,\;k\right)=\frac{MSR-MSE}{MSR+\left(k-1\right)MSE}$$

where 3 refers to the two-way mixed effect model, k is the number of raters, k=3 was set in this study, and MSR is the mean square for rows. When MSE=0, ICC(a, b)=1. ICC values less than 0.5 indicated poor reliability, values between 0.5 and 0.75 indicated moderate reliability, values between 0.75 and 0.9 indicated good reliability, and values greater than 0.90 indicated excellent reliability [36].

Bland–Altman plots were generated to evaluate the agreement of the MOS between operators in each image set. In the Bland–Altman plot, the horizontal axis shows the mean of the MOS between operators, and the vertical axis represents the difference in MOS between operators (*d*). Cohen’s weighted kappa (*k*) was used as a measure of agreement of interoperator variance [37,38]. This indicates the magnitude of the disagreement between the operators in the calculation. The interpretation of agreement for *k* was categorized as follows: Poor (*k* < 0), slight (0 ≤ *k* ≤ 0.2), fair (0.21 ≤ *k* ≤ 0.4), moderate (0.41 ≤ *k* ≤ 0.6), substantial (0.61 ≤ *k* ≤ 0.80), and almost perfect (*k* > 0.8).

The Wilcoxon signed-rank test was used for statistical analysis. Differences were considered statistically significant when *p* < 0.05. All statistical analyses were performed using the Statistical Package for the Social Sciences (SPSS) Statistics 26 (IBM Corp., Armonk, NY, USA).

## 4. Results

In the objective evaluation, the mean PSNR was 30.9 dB (95% confidence interval [CI]: 30.7–31.0 dB) in bilinear upsampling and 31.3 dB (95% CI: 31.1–31.5 dB) in the RDN model super-resolved image. In addition, the mean SSIM was 0.927 (95% CI: 0.924–0.930 dB) in bilinear upsampling and 0.931 (95% CI: 0.928–0.934 dB) in images super-resolved using the RDN model. Statistically significant differences were observed in both PSNR and SSIM (*p* < 0.05). Thus, the quality of the super-resolved image obtained using the RDN model was significantly better than that of the conventional bilinear upsampled image.

**Figure 2:**
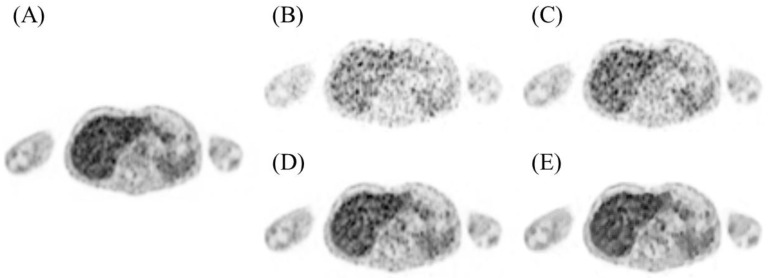
Representative images at liver level. (**A**) Original “ground-truth” image, (**B**) 10% PET image, (**C**) 20% PET image, (**D**) 50% PET image, and (**E**) super-resolved image obtained via a residual dense network from the 50% PET image.

**Figure 3:**
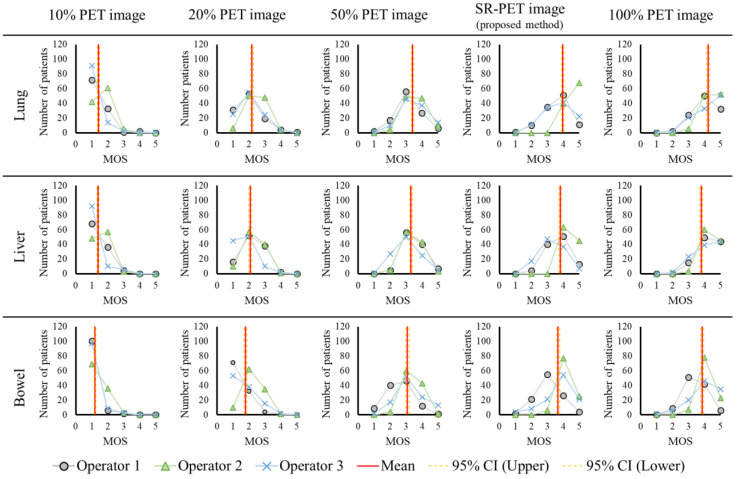
Subjective evaluation results of the MOS in each image set. PET: Positron emission tomography, SR-PET: Supe-resolved PET, MOS: Mean opinion score, and 95% CI: 95% confident interval.

**Figure 4:**
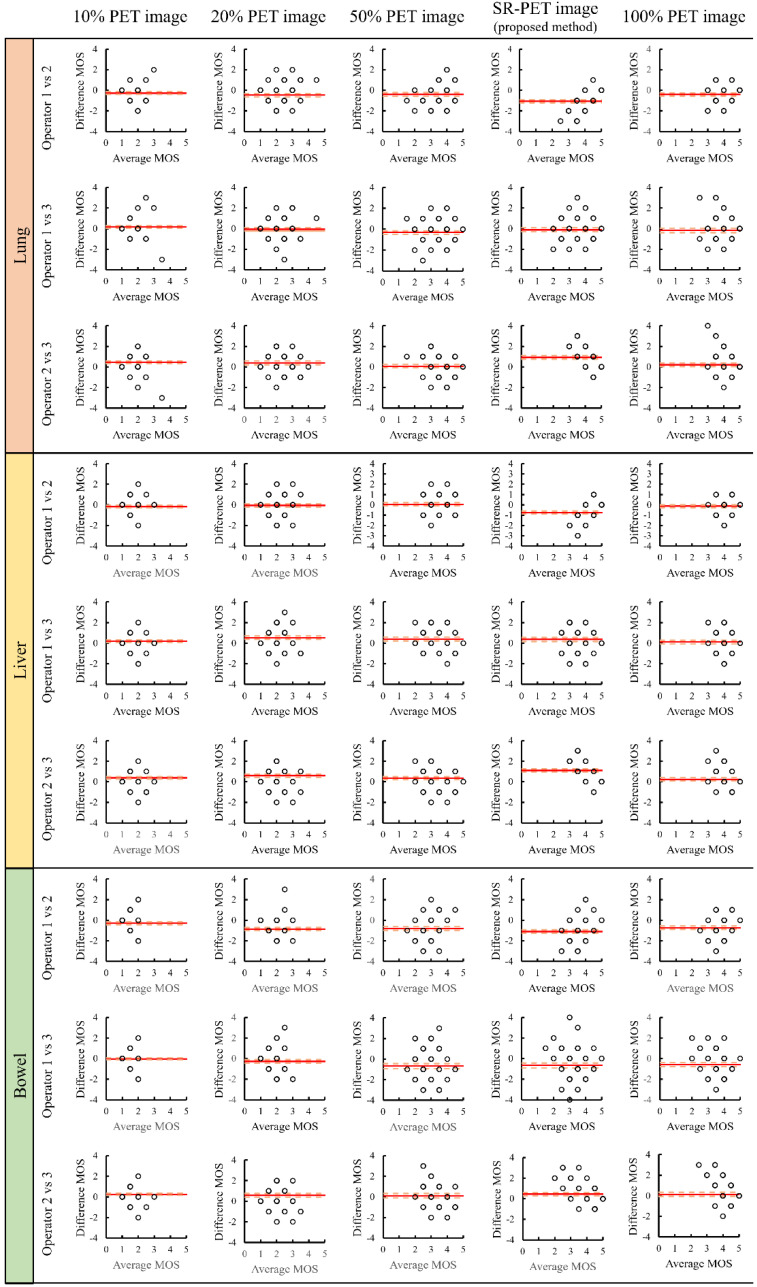
Bland–Altman plot showing the inter-operator difference in MOS for each image set. The red solid line denotes the mean of the difference, and the dashed line denotes the 95% limits of agreement. PET: Positron emission tomography, SR-PET: Super-resolved PET, MOS: Mean opinion score.

In the subjective evaluation, Figure 2 shows an example of an image at the liver level. Figure 3 shows the MOS results for each image set and Table 2 summarizes the subjective evaluation results. As shown in Table 2, the MOS of the super-resolved image obtained via the RDN model at all levels was significantly higher than that of the 50% PET image upsampled by the bilinear method (*p* < 0.05). However, the MOS of the super-resolved image obtained using the RDN model was significantly lower at all levels than that of the original image upsampled using the bilinear method (*p* < 0.05). Furthermore, Figure 4 shows the Bland–Altman plots for the inter-operator difference in MOS for each image set. The ICC estimates and their 95% CIs for the 50% PET image set were 0.62 (95% CI: 0.48–0.73) for the lung level, 0.56 (95% CI: 0.56–0.39) for the liver level, and 0.14 (95% CI: −0.18 to 0.39) for the bowel level. The ICC estimates and their 95% CIs for the SR-PET image set were 0.48 (95% CI: 0.28‒0.63) for the lung level, 0.43 (95% CI: 0.22‒0.60) for the liver level, and 0.32 (95% CI: 0.07–0.52) for the bowel level. All kappa indexes (*k*) exceeded 0.8 (Table 3).

## 5. Discussion

18F-FDG PET-CT scans are required to provide accurate tumor diagnoses and monitor the metabolic response of patients to treatment. However, this examination involves considerable radiation exposure [11,39]. By replacing CT with MRI, the resulting PET/MR system can reduce radiation exposure from CT scans. Many previous studies have focused on replacing CT with MRI for the registration of anatomical and functional information from PET. However, reduction of the injected FDG dose has received less attention. The major problem associated with a reduced injected FDG dose is the increase in image noise [21]. In this study, we evaluated the image quality of 18F-FDG PET images generated with 50% of the typically injected FDG dose. In this study, using the RDN model, we created an SR-PET image set from a 50% PET image obtained using a Vereos PET/CT scanner equipped with a SiPM detector. In our objective evaluation of PSNR and SSIM, the SR-PET image set showed high similarity to the conventional method. In addition, our subjective evaluation of MOS by three different experienced board-certified nuclear medicine physicians suggested that the SR-PET image set was of significantly higher quality than the 50% PET image set. On the other hand, the MOS of the SR-PET image set was significantly lower than that of the original “ground-truth” image. Moreover, the ICC was moderately reliable in all cases. Our results thus suggest that SR-PET images enable the use of a low FDG injection dose in whole-body PET scans. This is useful not only for adults, but also for pediatric and adolescent young adults.

Wang et al. demonstrated the generation of diagnostic 18F-FDG PET images of pediatric cancer patients from an ultra-low-dose (6.25%) 18F-FDG PET image by using a convoluted neural network algorithm [40]. Recently, image generation techniques, such as generative adversarial networks, have been considered for generating PET images, such as reconstructed normal injected doses from lower injected dose images [27,28]. Based on these reports, the use of simulated half-injected FDG dose images is reasonable [22,23]. The median injected FDG dose and exposure dose related to PET in clinical data were 258.7 MBq and 4.9 mSv (Table 1). If an SR image sufficient for diagnosis can be obtained from a half-acquisition-time PET image, it can be expected that the exposure dose will be reduced by half. Because a lower injected FDG dose for patients causes lower radiation exposure in PET scans, we should consider a much lower injected dose image as the input image set.

This study had some limitations. The first limitation is the selection of the SR deep-learning model architecture. As many new architectures are released every year, it was difficult to verify which model would be optimal for our purposes [2,3,4,5,6]. Therefore, we selected the RDN model, which achieved excellent results when considering this research plan. However, the RDN model was fine-tuned for natural-color images and not for medical grayscale images. However, since there are many colored quantitative images in medicine for evaluating blood flow and function [41,42] in the human body, training with three channels of RBG instead of one channel of grayscale input would be useful for transfer learning of the created model. Consequently, we converted the 16-bit DICOM image to an 8-bit PNG image, as the RDN model could not demonstrate its performance on PET images. As a result, the expressivity of the intensity histogram would be reduced. If an SR PET image was directly generated from the 16-bit DICOM image, the expressivity would maintain the intensity histogram. Recently, a model of direct super-resolution for DICOM images was proposed. Sim et al. designed deep convolutional networks for working with grayscale DICOM images of the brain [43]. In this way, further improvement can be expected by using the directly imported DICOM image model without needing to convert them to PNG images.

The second limitation of our study was inter-operator bias. As shown in Figure 4 and Table 3, the inter-operator agreement was almost perfect. However, the absolute *d* in our proposed method was higher than 1 at some points. This suggests that operator 2 understood the SR-PET images generated by the RDN model more than did the other operators.

The third limitation was the evaluation of the maximum standardized uptake value (SUV_max_). In this study, we mentioned the image quality of the SR PET image and did not sufficiently consider the quantitative diagnostic ability. As a quantitative measurement index of the de facto standard, SUV_max_ has been used to express the degree of FDG uptake. Normally, SUV_max_ is calculated using the DICOM tag with each voxel expressed in a 16-bit integer in the Metavol software package for PET-CT volumetric analysis [44,45]. Because quantitative measurements using SUV_max_ are important in clinical use, it is required to save the quantitative value for each voxel. In this study, however, the SUV_max_ was not calculated because we converted the 16-bit DICOM images into 8-bit PNG images. Therefore, the current challenge is to secure a quantitative value of SUV_max_ when using SR. Based on the above, we will improve the SR model and generate diagnostic PET images from much lower injected FDG doses in future research.

## 6. Conclusions

In conclusion, we evaluated the image quality of super-resolved images obtained from 50% simulated injected FDG-dose PET images using the RDN model. By using the SR model, the image quality was improved compared to that before SR image processing. Although there are some limitations to its clinical use, our results suggest that implementing the SR model could be effective in reducing the injected FDG dose for PET examination.

## Figures and Tables

**Table 1:** Patient characteristics and scan parameters in clinical data.

		Median	Range	
Age	(years)	66.5	20.0	86.0
BMI	(kg/m^2^)	22.4	12.3	34.7
Weight	(kg)	57.6	30.0	91.3
Height	(m)	1.60	1.40	1.78
Injection dose	(MBq)	258.7	124.0	400.7
Emission time	(s)	90	90	180
Uptake time	(min)	63	54	118
Exposure dose from PET	(mSv)	4.9	2.3	7.6
Number of images per patient		241	191	291

**Table 2:** Summary of the subjective evaluation of each image set.

		10% PET Image Set			20% PET Image Set			50% PET Image Set		
		Mean	(95% CI)		Mean	(95% CI)		Mean	(95% CI)	
MOS	Lung	1.41	1.35	1.48	2.17	2.09	2.26	3.41	3.32	3.49
	Liver	1.40	1.34	1.46	2.08	2.00	2.16	3.31	3.22	3.39
	Bowel	1.20	1.15	1.25	1.78	1.69	1.86	3.08	2.99	3.17
ICC (3,3)	Lung	0.48	0.28	0.63	0.53	0.36	0.67	0.62	0.48	0.73
	Liver	0.55	0.38	0.68	0.43	0.21	0.59	0.56	0.39	0.69
	Bowel	0.06	−0.29	0.33	0.35	0.10	0.54	0.14	−0.18	0.39
		**SR-PET Image** **(Proposed Method)**			**100% PET Image Set**					
		**Mean**	**(95% CI)**		**Mean**	**(95% CI)**				
MOS	Lung	3.96	3.87	4.05	4.23	4.15	4.31			
	Liver	3.80	3.71	3.89	4.27	4.20	4.35			
	Bowel	3.67	3.57	3.77	3.85	3.77	3.94			
ICC (3,3)	Lung	0.48	0.28	0.63	0.31	0.06	0.51			
	Liver	0.43	0.22	0.60	0.55	0.38	0.68			
	Bowel	0.32	0.07	0.52	0.19	−0.12	0.42			

PET: Positron emission tomography, SR-PET: Super-resolved PET, MOS: Mean opinion score, ICC: Intraclass correlation coefficients, and 95% CI: 95% confident interval.

**Table 3:** Summary of agreement of inter-operator variance using Cohen’s weighted kappa (*k*) statistics.

Operator		10% PET Image				20% PET Image				50% PET Image			
		*d*			*k*	*d*			*k*	*d*			*k*
		Mean	(95% CI)			Mean	(95% CI)			Mean	(95% CI)		
1 vs. 2	Lung	−0.28	−0.42	−0.14	0.96	−0.47	−0.64	−0.30	0.94 −0.40		−0.57	−0.23	0.94
	Liver	−0.18	−0.31	−0.05	0.97	−0.06	−0.22	0.11	0.95	0.05	−0.11	0.20	0.96
	Bowel	−0.30	−0.43	−0.17	0.97	−0.85	−0.99	−0.71	0.92 −0.79		−0.97	−0.61	0.91
1 vs. 3	Lung	0.18	0.04	0.31	0.97	−0.07	−0.26	0.11	0.94 −0.32		−0.50	−0.15	0.94
	Liver	0.20	0.08	0.32	0.97	0.53	0.36	0.69	0.94	0.40	0.22	0.57	0.94
	Bowel	−0.04	−0.14	0.06	0.98	−0.29	−0.47	−0.10	0.94 −0.68		−0.91	−0.44	0.88
2 vs. 3	Lung	0.45	0.32	0.59	0.96	0.40	0.23	0.57	0.94	0.07	−0.10	0.25	0.95
	Liver	0.38	0.26	0.50	0.96	0.58	0.41	0.76	0.93	0.35	0.19	0.51	0.95
	Bowel	0.26	0.14	0.38	0.97	0.56	0.38	0.75	0.92	0.11	−0.10	0.32	0.92
**Operator**		**SR-PET Image** **(Proposed Method)**				**100% PET Image**							
		***d***			***k***	***d***			***k***				
		**Mean**	**(95% CI)**			**Mean**	**(95% CI)**						
1 vs. 2	Lung	−1.06	−1.22	−0.91	0.86	−0.40	−0.56	−0.24	0.92				
	Liver	−0.74	−0.89	−0.59	0.93	−0.12	−0.26	0.01	0.97				
	Bowel	−1.09	−1.27	−0.92	0.87	−0.73	−0.89	−0.57	0.92				
1 vs. 3	Lung	−0.12	−0.32	0.08	0.93	−0.18	−0.39	0.04	0.92				
	Liver	0.36	0.17	0.56	0.93	0.11	−0.06	0.28	0.95				
	Bowel	−0.66	−0.89	−0.42	0.88	−0.58	−0.79	−0.38	0.91				
2 vs. 3	Lung	0.94	0.76	1.13	0.89	0.22	0.03	0.41	0.94				
	Liver	1.10	0.95	1.26	0.88	0.23	0.07	0.40	0.95				
	Bowel	0.44	0.25	0.62	0.93	0.15	−0.06	0.35	0.93				

*d*: Difference of MOS between operators, PET: Positron emission tomography, SR-PET: Super-resolved PET, MOS: Mean opinion score.

## Data Availability

Not applicable.

## References

[1] Dong C., Loy C.C., He K., Tang X. (2014). Learning a Deep Convolutional Network for Image Super-Resolution. Cham.

[2] Timofte R., Agustsson E., Gool L.V., Yang M.-H., Zhang L., Lim B., Son S., Kim H., Nah S., Lee K.M. NTIRE 2017 Challenge on Single Image Super-Resolution: Methods and Results. Proceedings of the IEEE Conference on Computer Vision and Pattern Recognition Workshops.

[3] Agustsson E., Timofte R. NTIRE 2017 Challenge on Single Image Super-Resolution: Dataset and Study. Proceedings of the IEEE Conference on Computer Vision and Pattern Recognition Workshops.

[4] Timofte R., Gu S., Wu J., Gool L.V., Zhang L., Yang M.H., Haris M., Shakhnarovich G., Ukita N., Hu S. NTIRE 2018 Challenge on Single Image Super-Resolution: Methods and Results. Proceedings of the 2018 IEEE/CVF Conference on Computer Vision and Pattern Recognition Workshops (CVPRW).

[5] Cai J., Gu S., Radu T., Lei Z. NTIRE 2019 Challenge on Real Image Super-Resolution: Methods and Results. Proceedings of the IEEE/CVF Conference on Computer Vision and Pattern Recognition (CVPR) Workshops 2019.

[6] Andreas L., Martin D., Radu T. NTIRE 2020 Challenge on Real-World Image Super-Resolution: Methods and Results. Proceedings of the IEEE/CVF Conference on Computer Vision and Pattern Recognition (CVPR) Workshops 2020.

[7] Browse State-of-the-Art. https://paperswithcode.com/sota.

[8] Hirata K., Sugimori H., Fujima N., Toyonaga T., Kudo K. (2022). Artificial intelligence for nuclear medicine in oncology. Ann. Nucl. Med..

[9] Farwell M.D., Pryma D.A., Mankoff D.A. (2014). PET/CT imaging in cancer: Current applications and future directions. Cancer.

[10] Sotoudeh H., Sharma A., Fowler K.J., McConathy J., Dehdashti F. (2016). Clinical application of PET/MRI in oncology. J. Magn. Reson. Imaging.

[11] Huang B., Law M.W.-M., Khong P.-L. (2009). Whole-Body PET/CT Scanning: Estimation of Radiation Dose and Cancer Risk. Radiology.

[12] Grant A.M., Deller T.W., Khalighi M.M., Maramraju S.H., Delso G., Levin C.S. (2016). NEMA NU 2-2012 performance studies for the SiPM-based ToF-PET component of the GE SIGNA PET/MR system. Med. Phys..

[13] Hsu D.F.C., Ilan E., Peterson W.T., Uribe J., Lubberink M., Levin C.S. (2017). Studies of a Next-Generation Silicon-Photomultiplier-Based Time-of-Flight PET/CT System. J. Nucl. Med..

[14] van Sluis J., de Jong J., Schaar J., Noordzij W., van Snick P., Dierckx R., Borra R., Willemsen A., Boellaard R. (2019). Performance Characteristics of the Digital Biograph Vision PET/CT System. J. Nucl. Med..

[15] Rausch I., Ruiz A., Valverde-Pascual I., Cal-Gonzalez J., Beyer T., Carrio I. (2019). Performance Evaluation of the Vereos PET/CT System According to the NEMA NU2-2012 Standard. J. Nucl. Med..

[16] Zimmermann P.A., Houdu B., Cesaire L., Nakouri I., De Pontville M., Lasnon C., Aide N. (2021). Revisiting detection of in-transit metastases in melanoma patients using digital (18) F-FDG PET/CT with small-voxel reconstruction. Ann. Nucl. Med..

[17] Alberts I., Sachpekidis C., Prenosil G., Viscione M., Bohn K.P., Mingels C., Shi K., Ashar-Oromieh A., Rominger A. (2021). Digital PET/CT allows for shorter acquisition protocols or reduced radiopharmaceutical dose in [(18).F]-FDG PET/CT. Ann. Nucl. Med..

[18] Japan Network for Research and Information on Medical Exposure (J-RIME), National Diagnostic Reference Levels in Japan (2020)-Japan DRLs 2020. http://www.radher.jp/J-RIME/report/DRL2020_Engver.pdf.

[19] Abe K., Hosono M., Igarashi T., Iimori T., Ishiguro M., Ito T., Nagahata T., Tsushima H., Watanabe H. (2020). The 2020 national diagnostic reference levels for nuclear medicine in Japan. Ann. Nucl. Med..

[20] Boellaard R., Delgado-Bolton R., Oyen W.J., Giammarile F., Tatsch K., Eschner W., Verzijlbergen F.J., Barrington S.F., Pike L.C., Weber W.A. (2015). FDG PET/CT: EANM procedure guidelines for tumour imaging: Version 2.0. Eur. J. Nucl. Med. Mol. Imaging.

[21] Karakatsanis N.A., Fokou E., Tsoumpas C. (2015). Dosage optimization in positron emission tomography: State-of-the-art methods and future prospects. Am. J. Nucl. Med. Mol. Imaging..

[22] Queiroz M.A., Delso G., Wollenweber S., Deller T., Zeimpekis K., Huellner M., de Galiza Barbosa F., von Schulthess G., Veit-Haibach P. (2015). Dose Optimization in TOF-PET/MR Compared to TOF-PET/CT. PLoS ONE.

[23] Sekine T., Delso G., Zeimpekis K.G., de Galiza Barbosa F., Ter Voert E., Huellner M., Veit-Haibach P. (2018). Reduction of (18)F-FDG Dose in Clinical PET/MR Imaging by Using Silicon Photomultiplier Detectors. Radiology.

[24] Dilsizian V., Bacharach S.L., Beanlands R.S., Bergmann S.R., Delbeke D., Dorbala S., Gropler R.J., Knuuti J., Schelbert H.R., Travin M.I. (2016). ASNC imaging guidelines/SNMMI procedure standard for positron emission tomography (PET) nuclear cardiology procedures. J. Nucl. Cardiol..

[25] Ooi Y.K., Ibrahim H. (2021). Deep Learning Algorithms for Single Image Super-Resolution: A Systematic Review. Electronics.

[26] Ledig C., Theis L., Huszar F., Caballero J., Cunningham A., Acosta A., Aitken A., Tejani A., Totz J., Wang Z. Photo-Realistic Single Image Super-Resolution Using a Generative Adversarial Network. Proceedings of the IEEE Conference on Computer Vision and Pattern Recognition Workshops.

[27] Lei Y., Dong X., Wang T., Higgins K., Liu T., Curran W.J., Mao H., Nye J.A., Yang X. (2019). Whole-body PET estimation from low count statistics using cycle-consistent generative adversarial networks. Phys. Med. Biol..

[28] Ouyang J., Chen K.T., Gong E., Pauly J., Zaharchuk G. (2019). Ultra-low-dose PET reconstruction using generative adversarial network with feature matching and task-specific perceptual loss. Med. Phys..

[29] Radiation Dose to Patients from Radiopharmaceuticals (Addendum to ICRP Publication 53) (1998). ICRP Publication 80. Ann. ICRP.

[30] Zhang Y., Tian Y., Kong Y., Zhong B., Fu Y. Residual Dense Network for Image Super-Resolution. Proceedings of the IEEE Conference on Computer Vision and Pattern Recognition.

[31] Vabalas A., Gowen E., Poliakoff E., Casson A.J. (2019). Machine learning algorithm validation with a limited sample size. PLoS ONE.

[32] Hore A., Ziou D. Image quality metrics: PSNR vs. SSIM. Proceedings of the 2010 20th International Conference on Pattern Recognition.

[33] Wang Z., Bovik A.C., Sheikh H.R., Simoncelli E.P. (2004). Image quality assessment: From error visibility to structural similarity. IEEE Trans. Image Process..

[34] Preedy V.R., Watson R.R., 5-Point Likert Scale (2010). Handbook of Disease Burdens and Quality of Life Measures.

[35] Müller R., Büttner P. (1994). A critical discussion of intraclass correlation coefficients. Stat. Med..

[36] Koo T.K., Li M.Y. (2016). A Guideline of Selecting and Reporting Intraclass Correlation Coefficients for Reliability Research. J. Chiropr. Med..

[37] Fleiss J.L., Cohen J. (1973). The Equivalence of Weighted Kappa and the Intraclass Correlation Coefficient as Measures of Reliability. Educ. Psychol. Meas..

[38] Landis J.R., Koch G.G. (1977). The Measurement of Observer Agreement for Categorical Data. Biometrics.

[39] Brenner D.J., Hall E.J. (2007). Computed Tomography—An Increasing Source of Radiation Exposure. N. Engl. J. Med..

[40] Wang Y.J., Baratto L., Hawk K.E., Theruvath A.J., Pribnow A., Thakor A.S., Gatidis S., Lu R., Gummidipundi S.E., Garcia-Diaz J. (2021). Artificial intelligence enables whole-body positron emission tomography scans with minimal radiation exposure. Eur. J. Nucl. Med. Mol. Imaging.

[41] Fahlstrom M., Appel L., Kumlien E., Danfors T., Engstrom M., Wikstrom J., Antoni G., Larsson E.M., Lubberink M. (2021). Evaluation of Arterial Spin Labeling MRI-Comparison with (15) O-Water PET on an Integrated PET/MR Scanner. Diagnostics.

[42] Le Roux P.Y., Hicks R.J., Siva S., Hofman M.S. (2019). PET/CT Lung Ventilation and Perfusion Scanning using Galligas and Gallium-68-MAA. Semin. Nucl. Med..

[43] Sim K.S., Sammani F. (2019). Deep convolutional networks for magnification of DICOM brain images. Int. J. Innov. Comput. Inf. Control..

[44] Hirata K., Manabe O., Magota K., Furuya S., Shiga T., Kudo K. (2021). A Preliminary Study to Use SUVmax of FDG PET-CT as an Identifier of Lesion for Artificial Intelligence. Front Med. Lausanne.

[45] Hirata K., Kobayashi K., Wong K.P., Manabe O., Surmak A., Tamaki N., Huang S.C. (2014). A semi-automated technique determining the liver standardized uptake value reference for tumor delineation in FDG PET-CT. PLoS ONE.

